# Diffusion Tensor Imaging-Based Research on Human White Matter Anatomy

**DOI:** 10.1100/2012/530432

**Published:** 2012-11-25

**Authors:** Ming-guo Qiu, Jing-na Zhang, Ye Zhang, Qi-yu Li, Bing Xie, Jian Wang

**Affiliations:** ^1^Department of Medical Informatics and Medical Image, College of Biomedical Engineering and Medical Imaging, Third Military Medical University, Chongqing 400038, China; ^2^Department of Anatomy, Third Military Medical University, Chongqing 400038, China; ^3^Department of Radiology, Southwest Hospital, Third Military Medical University, Chongqing 400038, China

## Abstract

The aim of this study is to investigate the white matter by the diffusion tensor imaging and the Chinese visible human dataset and to provide the 3D anatomical data of the corticospinal tract for the neurosurgical planning by studying the probabilistic maps and the reproducibility of the corticospinal tract. Diffusion tensor images and high-resolution T1-weighted images of 15 healthy volunteers were acquired; the DTI data were processed using DtiStudio and FSL software. The FA and color FA maps were compared with the sectional images of the Chinese visible human dataset. The probability maps of the corticospinal tract were generated as a quantitative measure of reproducibility for each voxel of the stereotaxic space. The fibers displayed by the diffusion tensor imaging were well consistent with the sectional images of the Chinese visible human dataset and the existing anatomical knowledge. The three-dimensional architecture of the white matter fibers could be clearly visualized on the diffusion tensor tractography. The diffusion tensor tractography can establish the 3D probability maps of the corticospinal tract, in which the degree of intersubject reproducibility of the corticospinal tract is consistent with the previous architectonic report. DTI is a reliable method of studying the fiber connectivity in human brain, but it is difficult to identify the tiny fibers. The probability maps are useful for evaluating and identifying the corticospinal tract in the DTI, providing anatomical information for the preoperative planning and improving the accuracy of surgical risk assessments preoperatively.

## 1. Introduction

 The study of the brain white matter is an important and also a difficult part of the research on the human brain function and structure; the invasional fiber tracking methods used in the animals cannot be used in the human beings [1–3]. It is difficult to display the three-dimensional structure of the brain white matter by gross anatomy or sectional anatomy, and so it is to segment the white matter in the Chinese Visible Human dataset [4]. 

Diffusion tensor magnetic resonance imaging (DT-MRI) is a valuable tool for studying the microstructure of the brain in vivo and has the ability to reveal microstructural properties of the white matter [5–7]. It has been shown that, in the brain, ordered axonal structure, cell membrane, and myelin sheath strongly influence water diffusion and that there is a direct link between water diffusion and axonal orientation and integrity. In fact, when diffusion tensor imaging (DTI) is performed within a compact tract with parallel running axonal trajectories like the corticospinal tract, the DT is strongly anisotropic and its principal eigenvector corresponds to the direction of the fiber tract [8, 9]. DTI has been used extensively in the study of the brain white matter; it can display the three-dimensional structure of the brain white matter and can quantitatively analyze the special fiber [10–12]. 

DTI has opened up new opportunities for analyzing the position and extent of individual fiber tracts in vivo using diffusion tensor tractography [13, 14]. The aim of the present study was to investigate the reliability of the diffusion tensor imaging by comparing it with the Chinese visible human dataset, to display the three-dimensional architecture of the white matter fibers in human brain by using diffusion tensor tractography, and to provide the 3D anatomical data of the corticospinal tract for the neurosurgical planning by studying the probabilistic maps and the reproducibility of the corticospinal tract.

## 2. Methods

### 2.1. Subjects

Fifteen healthy volunteers (aged 22–39, 9 males and 6 females) were recruited as the subjects of an ongoing functional MR imaging study. Volunteers had no history of a psychiatric disease or a neurological injury. An informed consent from all subjects was obtained in accordance with institutional guidelines. 

### 2.2. Image Acquisition

DTI data were acquired using dual spin-echo, single shot echo-planar imaging sequence on a 3.0T Siemens Sonata scanner with 3-mm slice thickness, no interslice gap, TR=6400 ms, TE=88 ms, FOV=220×220 mm, 12 noncollinear diffusion-sensitizing gradient directions with diffusion sensitivity *b* = 1000 s/mm^2^, 6 averages, matrix = 128 × 128, and 40 contiguous slices yielding full brain coverage. Slices were positioned along the anterior commissure-posterior commissure line. A normalizing T2 image without diffusion weighting was also acquired. Immediately after the EPI scan, a whole-brain high-resolution T1-weighted anatomical image was acquired with 3D magnetization-prepared rapid acquisition gradient echo (MPRAGE) sequence, TR/TE/FA = 2300 ms/3.93 ms/8°, matrix = 256 × 256, and 1 mm slice thickness. 

### 2.3. Image Processing and Analysis

Firstly, DTI data were processed with DtiStudio software [15] to reconstruct the FA, and the color FA maps. From the sectional images of the CVH dataset [4], the transversal, coronal, and sagittal images of the head (thickness = 0.1 mm) were selected. The reconstructed FA and color FA maps were compared with the sectional images of the CVH dataset, to define the position and the contour of the fiber tract.

Secondly, the white matter fibers in the brain were reconstructed using DtiStudio tractography, in order to visualize anatomical connections in the form of separate identifiable tracts or bundles. In a deterministic framework [15, 16], we defined a region of interest (ROI) as a volume that selects fibres. The placement of the ROI was chosen according to the structure that was investigated and guided by the knowledge coming from postmortem anatomical studies and the CVH dataset. If necessary, a second (and even a third) ROI would be selected, in order to separate the tract of interest from others. The different tracts selected were identified by confrontation with the postmortem studies.

Then the DTI data were analyzed using FSL 4.1 (FMRIB Software Library, Oxford, UK). Preprocessing of DTI data included eddy current correction and computations of the diffusion tensor elements and FA maps [6]. The masks were outlined with blue color at the peduncle and the posterior limb of the internal capsule in the FA color maps using FSLView. In thecolor FA maps, red, green, and blue colors were assigned to the right-left, anterior-posterior, and superior-inferior orientations, respectively.T1 images were registered to the ICBM152-T1 template, and then the diffusion space was transferred to the standard space, with the masks serving as the seeds and the waypoints for probabilistic tractography. The probability maps were generated as a quantitative measure of reproducibility for each voxel of the stereotaxic space.

## 3. Results

### 3.1. Comparing the FA and Color FA Maps with the Sections of CVH

The major fibers of the white matter can be identified on the FA maps and the color FA maps. In thecolor FA maps, the red, green, and blue colors were assigned to the right-left, anterior-posterior, and superior-inferior orientations, respectively. Comparing the FA maps and the color FA maps with the sections of CVH, we found that the white matter displayed by the FA maps and the color FA maps were well consistent with the sectional images of the CVH dataset and the known anatomy, but it is difficult to identify the tiny fibers in the FA maps and the color FA maps.

**Figure 1:**
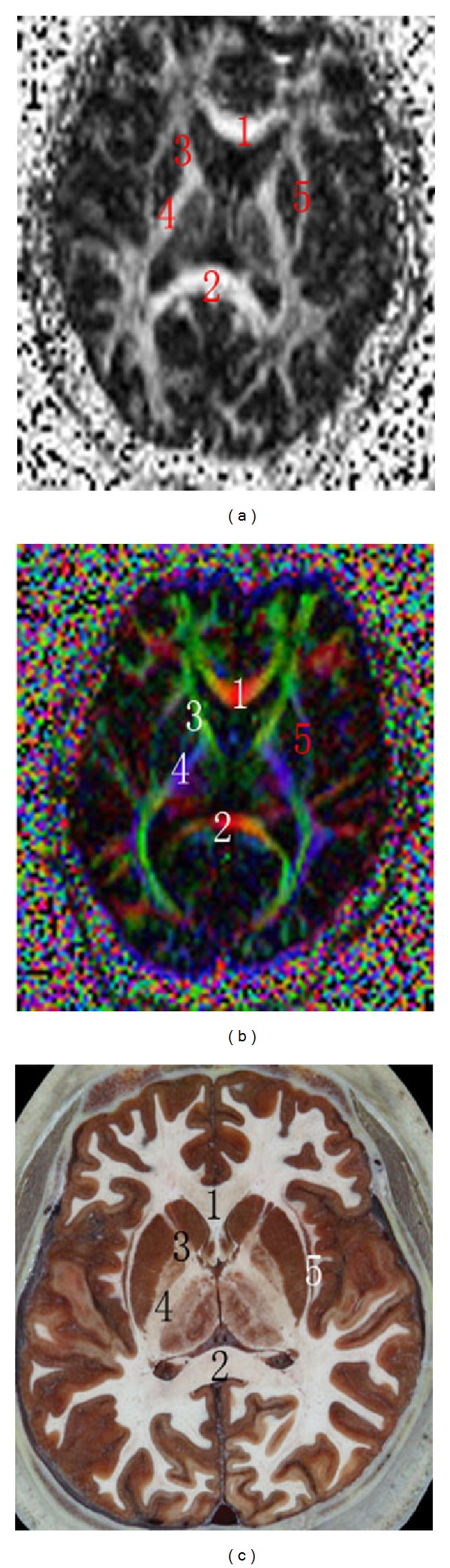
Transversal FA map, color tensor map, and sectional image of visible human at basal nuclei. (1) Genu of corpus callosum, (2) splenium of corpus callosum, (3) anterior limb of internal capsule, (4) posterior limb of internal capsule, and (5) external capsule and extreme capsule.

On the transversal FA map, color FA map, and sectional image of visible human at basal nuclei, the genu of corpus callosum and the splenium of corpus callosum were red, the anterior limb of internal capsule was green, and the posterior limb of internal capsule was blue. The external capsule and extreme capsule were mixed and could not be differentiated on the FA map or the color FA map, but were clear in the sectional image of the CVH (Figure 1).

**Figure 2:**
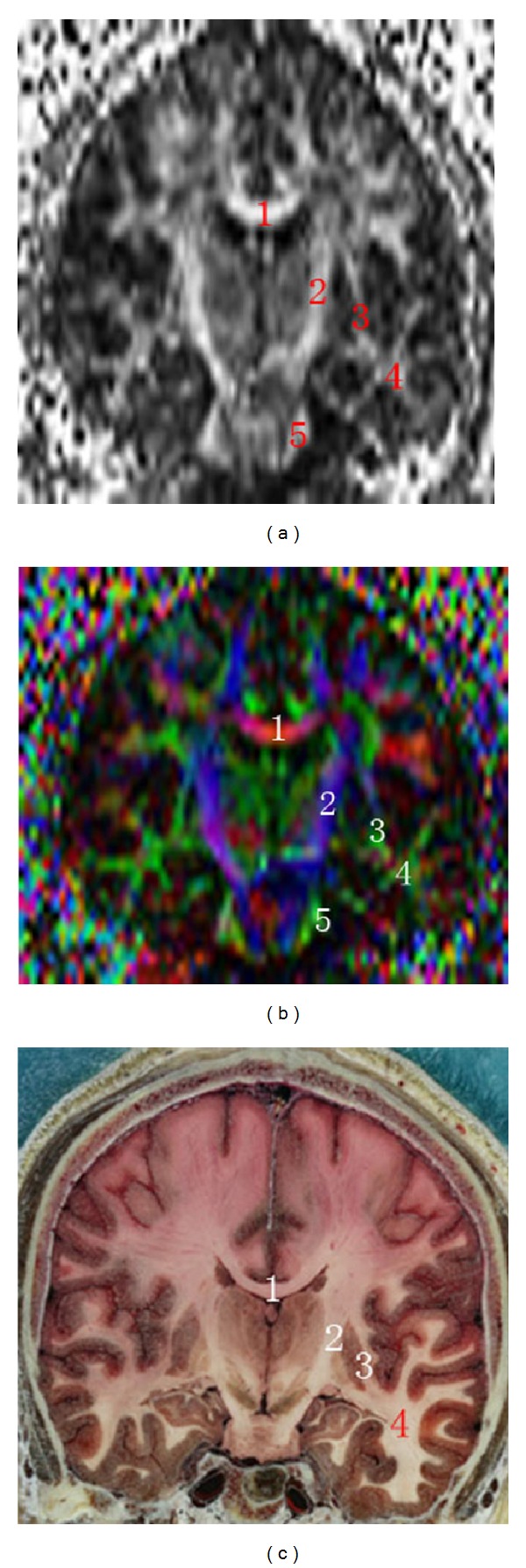
Coronal FA map, color tensor map, and sectional image of visible human at the posterior limb of internal capsule. (1) Body of corpus callosum, (2) pyramidal tract, (3) external capsule and extreme capsule, (4) inferior longitudinal fasciculus, and (5) middle cerebellar peduncle.

**Figure 3:**
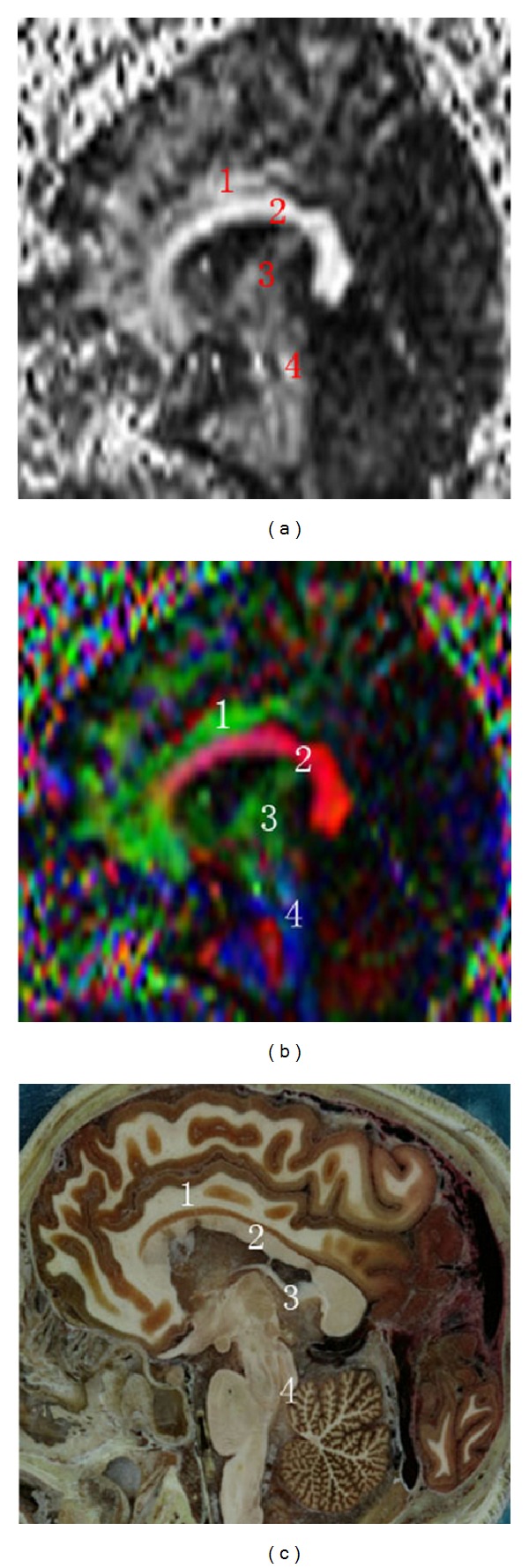
Sagittal FA map, color tensor map, and sectional image of visible human at the fornix. (1) Cingulate fasciculus, (2) corpus callosum, (3) fornix, and (4) superior cerebellar peduncle.

The coronal FA map, color FA map, and sectional image of visible human at the posterior limb of internal capsule displayed clearly the body of corpus callosum, the pyramidal tract, and the external capsule and the extreme capsule, but the fibers in the temporal lobe cannot be displayed clearly in the coronal FA map or the color FA map (Figure 2). In the sagittal FA map, color tensor map, and sectional image of a visible human at the fornix, we can see the cingulate fasciculus, the corpus callosum, the fornix, and the superior cerebellar peduncle (Figure 3).

### 3.2. Tractography of the White Matter

We adopted the above-mentioned methodology in order to perform a virtual dissection of several well-known anatomical systems and displayed the tract of interest on 3D views. The fibers displayed by diffusion tensor tractography were well consistent with the known anatomy. 

**Figure 4:**
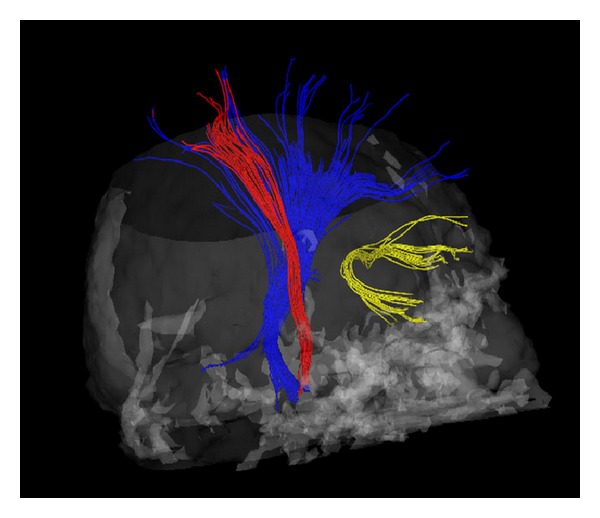
Spatial relation of the pyramidal tract (red), the medial lemniscus (blue), and the uncinate fasciculus (yellow).

**Figure 5:**
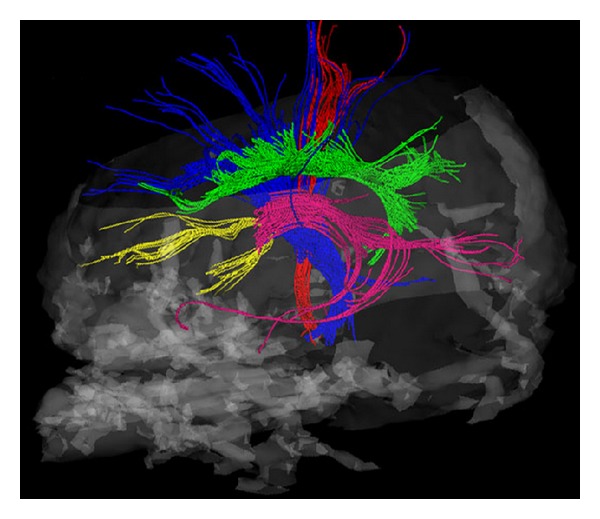
Spatial relation of the cingulated fasciculus (green), the fornix (purple), the uncinate fasciculus (yellow), the medial lemniscus (blue), and the pyramidal tract (red).

The 3D architecture of white matter fibers could be clearly visualized by diffusion tensor tractography, including the projection fibers of the pyramidal tract, the visual radiation, and the medial lemniscuses (Figures 4 and 5). The corticospinal tract is a large, well-characterized, and highly anisotropic tract. The ROI was placed more laterally in the ventrolateral part of the cerebral peduncle, and the selected fibres corresponded to the pyramidal tract, originating mainly from the region of the central sulcus and travelling down the brain stem (Figure 4). Looking at the color FA map at the level of the pons, we could easily identify the medial lemniscus and select the first ROI. Another ROI was placed in the anterior limb of the internal capsule (Figures 4 and 5).

**Figure 6:**
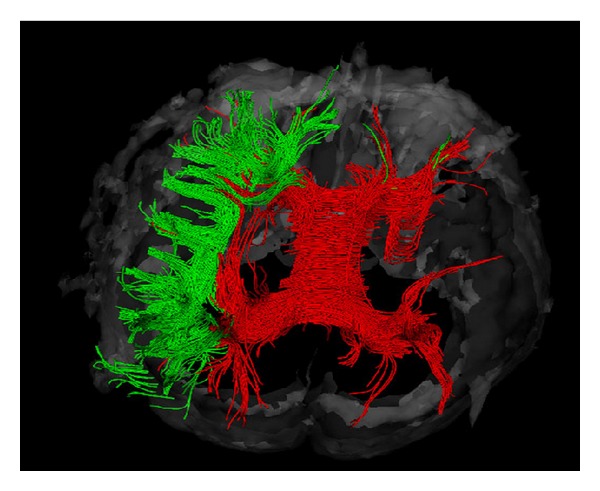
Spatial relation of the superior longitudinal fasciculus and the *U*-shaped fibers (green) to the corpus callosum (red).

The commissural fibers include the corpus callosum and the fornix (Figures 5 and 6). In order to identify the fibres passing through the corpus callosum, we placed a large ROI encompassing the whole corpus callosum at the midsagittal plane (Figure 6). Some of the major pathways constituting the limbic system are known to be the fornix and the cingulate bundle. The horizontal portion of the fornix was isolated by placing an ROI in a frontal plane parasagittally beneath the body of the corpus callosum.

**Figure 7:**
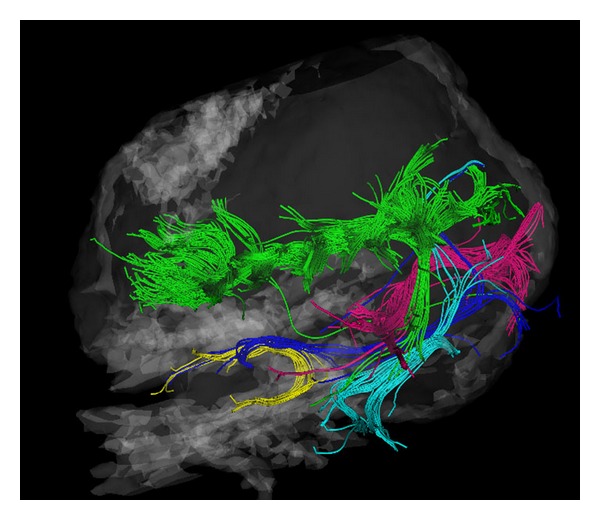
Spatial relation of the superior longitudinal fasciculus (green), the inferior longitudinal fasciculus (cyan), the optic radiation (purple), the uncinate fasciculus (yellow), and the inferior frontooccipital fasciculus (blue).

The association fibers include the uncinate fascicules, the cingulum and the superior and inferior longitudinal fasciculi (Figures 4 and 7). To identify the uncinate fascicle, we placed the first ROI in the anterior part of the temporal lobe and the second one in the frontal lobe (Figure 4). The cingulum was identified by using two ROIs placed in a frontal plane, 2 cm apart within the white matter of the cingulate gyrus (Figure 5). Corticocortical connection *U*-shaped fibers were widespread and formed only loose association bundles that are variable in size and shape (Figure 6). Figures 6 and 7 show the spatial relation of the superior longitudinal fasciculus and the *U*-shaped fibers to the corpus callosum; Figure 7 shows the inferior frontooccipital fasciculus as it was isolated by the two ROIs. The first was placed in the posterior parietal and the second in the frontal lobe. The superior longitudinal fascicle was selected by two ROIs placed below the motor and the posterior parietal cortices in frontal planes. In order to isolate the inferior longitudinal fascicle, we also used two selection ROIs. The posterior was, as for the inferior occipitofrontal fascicle, in the posterior parietal lobe, whereas the second was in the temporal lobe.

### 3.3. Probability Maps of the Corticospinal Tract

**Figure 8:**
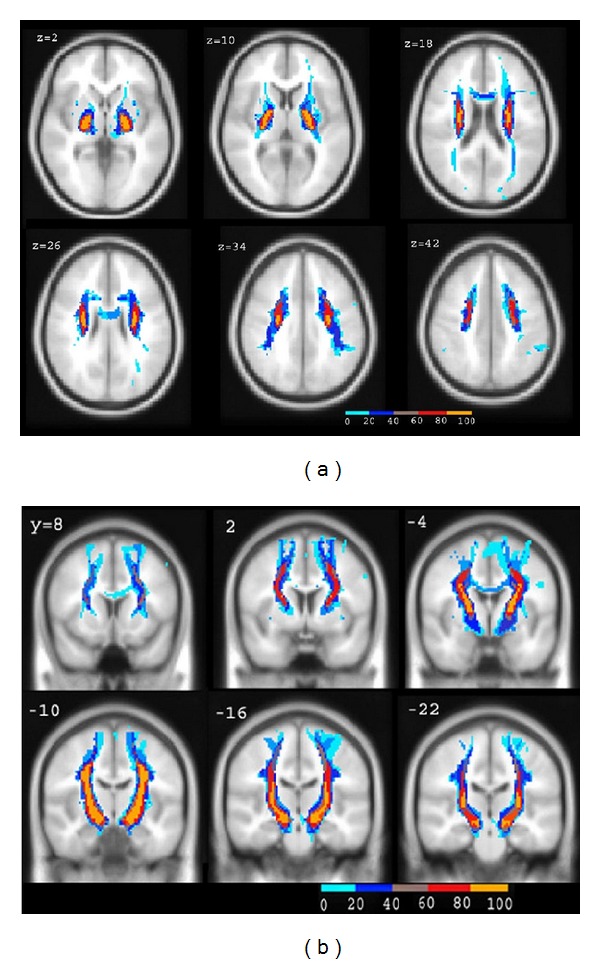
The corticospinal tract overlapped in axial sections (a) and coronal sections (b). The color bars indicate the relative frequency of voxels containing the corticospinal tract from 0 to 100% (yellow) at the 20% step.

In the probability maps of the corticospinal tract showing the axial sections and the coronal sections in the ICBM152-T1 template, the color bars indicate the relative frequency of voxels containing the corticospinal tract from 0 to 100% (yellow) at the 20% step (Figure 8). The coordinates of the corticospinal tract in the anatomical MNI space (orientation according to the AC-PC line) showed that *x* was from −58 to 1 in the left and from 2 to 54 in the right, *y* was from 20 to −56 in the left and from 12 to −51 in the right, and *z* was from −20 to 80 in the left and from −20 to 76 in the right. The mean volume of the left corticospinal tract was (57416.67 ± 1944.56) mm^3^ and that of the right corticospinal tract was (54421.58 ± 1232.43) mm^3^. The degree of the intersubject reproducibility shifted along its course with the highest reproducibility in the internal capsule, and the reproducibility of the corticospinal tract was higher in the right hemisphere than that in the left hemisphere. 

## 4. Discussion

The white matter displayed by the FA maps and the color FA maps were well consistent with that in the sectional images of the CVH dataset and the existing anatomical knowledge [1, 2, 4]. But the DTI is insufficient to display the tiny white matter fiber such as the external capsule and the extreme capsule and the fibers in the temporal lobe. On the other hand, it is very difficult to identify and segment the white matter fibers in the CVH dataset [4]. With the different fibers crossing together, how to identify, segment, and reconstruct each fiber? It is difficult to solve the problem using the current CVH dataset and the image postprocessing technique. However, we can acquire the DTI data of the healthy subjects; the major fibers of the white matter can be identified in the FA maps. And in thecolor FA maps, the red, green, and blue colors were assigned to right-left, anterior-posterior, and superior-inferior orientations, respectively. These cannot be achieved by the previous imaging technique. Thus, the DTI provides more information than the conventional MRI. By fusing the DTI data with the CVH dataset, we can make up for each other's deficiencies so as to enrich the content and enhance the function of the current CVH dataset, to observe directly the relationship between the anatomical structure and the white matter fibers, and to extend the application of the CVH dataset in the clinic.

The three-dimensional architecture of the white matter fibers could be clearly visualized with the diffusion tensor tractography, including the projection fibers, the commissural fibers, and the association fibers. The results showed striking similarity with the existing anatomical knowledge; the position and the extent of the fibers were consistent with the previous studies [2, 17]. Our results suggest that the fibre tracking is a valuable way for mapping the brain white matter. But it is necessary to note that, only the largest and the most homogeneous fibre bundles, which are not smaller than a voxel in diameter, can be followed [6, 7]. Using knowledge-based ROI for tract selection is not an ideal solution. A risk of biasing the results remains if the windows are not placed fairly. On the other hand, it is very important to master the anatomy of the white matter fiber, especially the sectional anatomy of the fiber, with the purpose of identifying the fiber on FA and color FA maps, and placing the ROI accurately. 

Another remaining problem for fibre tracking is the limited resolution of the imaging scanner and the incapacity of a tensor to model properly multiple fibre tracts in one voxel [13, 18]. Current deterministic streamlining algorithms follow the main eigenvector of the diffusion tensor, reducing the available information. Thus, only the largest and the most homogeneous fibre bundles, which are not smaller than a voxel in diameter, can be followed. This fibre tracking method has the following limitations: firstly, the diffusion tensor maybe is the mixture of multiple fiber diffusion. In some areas, such as the stem of the largest and the most homogeneous fiber bundles or the fibers bigger than a voxel in diameter, the main eigenvector of the diffusion tensor can be thought as the direction of the local fiber tracking. But in areas where the fibers cross, converge, or depart from each other, the main eigenvector of the voxel is the mean diffusion tensor of the different mixed fibers, it is obviously wrong to take the direction of the local fiber tracking as the main eigenvector [13, 19]. If the eigenvector that has the biggest diffusion tensor replaces the mean eigenvector of the voxel, the reconstruction of the other fibers will be restricted. Secondly, the noise and the regional volume effect will induce the fiber tracking error. Because of the limited resolution of the diffusion imaging, the effect of the imaging scanner, and the other noise during the images acquiring, it will induce an unreal result and add the fiber tracking error. The streamlining algorithm can only display the largest and the most homogeneous fibre bundles and is difficult to display the crossing fiber and those in subcortex or near cortex, so it will underestimate the volume of the fiber, even miscalculate the direction of the fiber.

The variety of the brain white matter can provide the very important anatomical information, providing valuable morphological information for the study of the disease in the nervous system and the neurosurgical planning [14, 20, 21]. The DTI probabilistic tractography has opened up new opportunities for analyzing the position and the extent of individual fiber tracts in vivo. The probabilistic tractography is to analyze the direction in each voxel [6]. Behrens studied the connectivity of the brain by using probabilistic tractography firstly, which can analyze the fiber in the subcortical or near the cortex, including the thalamus and the language function area [6, 22]. Our results showed that the volume of corticospinal tract is bigger than that of previous results, that is maybe because of the more accurate probabilistic tractography [14, 20, 21]. The diffusion tensor probabilistic tractography can establish the 3D probability maps of the corticospinal tract, and the degree of intersubject reproducibility of the corticospinal tract is consistent with previous architectonic report [1, 2]. The maps are useful for evaluating and identifying the corticospinal tract in DTI, providing anatomical information for preoperative planning and improving the accuracy of surgical risk assessments preoperatively. It is valuable to establish the probabilistic maps of the major fiber tracts by diffusion tensor probabilistic tractography.
